# Compound K Promotes Megakaryocytic Differentiation by NLRP3 Inflammasome Activation

**DOI:** 10.3390/biom14101257

**Published:** 2024-10-04

**Authors:** Seonhwa Hwang, Min-Seo Park, Anthony Junhoe Koo, Eunsoo Yoo, Seh-Hyon Song, Hye-Kyung Kim, Min-Hi Park, Jae-Seon Kang

**Affiliations:** 1 College of Pharmacy, Kyungsung University, 309 Suyeong-ro, Busan 48434, Republic of Korea; reseon17@naver.com (S.H.); minseo829@naver.com (M.-S.P.); shsong@ks.ac.kr (S.-H.S.); fiona30@ks.ac.kr (H.-K.K.); 2 Brain Busan 21 Plus Research Project Group, Kyungsung University, Busan 48434, Republic of Korea; 3 College of Engineering, North Carolina A&T State University, Greensboro, NC 27411, USA; junhoekoo0727@gmail.com; 4 Chemical, Biological, and Bioengineering Department, North Carolina A&T State University, Greensboro, NC 27411, USA; eyoo@ncat.edu

**Keywords:** compound K, megakaryocytic differentiation, leukemia cell, NLRP3 inflammasome, platelets

## Abstract

Platelets are essential blood components that maintain hemostasis, prevent excessive bleeding, and facilitate wound healing. Reduced platelet counts are implicated in various diseases, including leukemia, hepatitis, cancer, and Alzheimer’s disease. Enhancing megakaryocytic differentiation is a promising strategy to increase platelet production. Compound K (CK), a major bioactive metabolite of ginsenosides from *Panax ginseng*, has demonstrated anti-cancer and neuroprotective properties. In this study, we investigated the effects of CK on megakaryocytic differentiation and apoptosis in chronic myeloid leukemia (CML) cell lines K562 and Meg-01. CK treatment significantly upregulated the mRNA expression of key megakaryocytic differentiation markers, including CD61, CD41, and CD42a, and promoted the formation of large, multinucleated cells in K562 cells. Additionally, flow cytometry analysis revealed that CK at 5 µM induced apoptosis, a critical process in thrombocytopoiesis, in both K562 and Meg-01 cells. RT^2^ Profiler PCR array analysis further identified a marked increase in the expression of genes associated with the activation of the NLRP3 inflammasome in CK-treated K562 and Meg-01 cells. This study is the first to demonstrate that CK promotes megakaryocytic differentiation and apoptosis through the activation of the ERK/EGR1 and NLRP3 inflammasome pathways. These findings suggest that CK may enhance platelet production, indicating its potential as a therapeutic candidate for platelet-related disorders and other associated diseases.

## 1. Introduction

Platelets, also known as thrombocytes, are indispensable blood cells involved in a range of physiological processes, including hemostasis, wound healing, inflammation, tumor metastasis, and host defense [1,2,3]. These small, disk-shaped, anucleate cells originate from megakaryocytes (MKs), which are primarily located in the lungs and bone marrow [4,5]. MKs are derived from hematopoietic stem cells (HSCs) and undergo a complex sequence of regulated processes—such as DNA endoreduplication, cytoplasmic maturation, and cellular expansion—to produce and release platelets into circulation [6,7,8,9]. Although MKs are relatively rare in the bone marrow, they play a critical role in hematopoiesis and the regulation of hemostasis, ensuring the continuous supply of platelets necessary for maintaining key physiological functions [6,7,8].

Megakaryopoiesis, the process by which MKs develop from HSCs, is primarily regulated by thrombopoietin (TPO), a key hormone that promotes megakaryocyte (MK) colony formation and stimulates the proliferation and maturation of MK progenitors [10,11]. TPO binds to c-Mpl receptors on MKs and platelets, triggering a critical signal transduction cascade essential for MK development. Additionally, cytokines such as interleukin (IL)-1β, IL-3, and IL-6 contribute to the regulation of MK proliferation and maturation, often acting synergistically with TPO to enhance these processes [12]. As MKs progress through maturation, they undergo profound structural changes, accumulating internal membrane systems, granules, and organelles in preparation for platelet biogenesis. MK differentiation is marked by a shift from mitotic division to polyploidization via endomitosis, a process that increases both cell size and ploidy levels, ultimately enabling a single MK to produce thousands of platelets [7,13].

Platelets play a role beyond their traditional function in hemostasis, being actively involved in various physiological and pathological processes such as inflammation, immune responses, and tumor metastasis [14,15,16]. Decreased platelet counts have been linked to a range of diseases, including leukemia, hepatitis, cancer, and Alzheimer’s disease (AD) [17,18]. Moreover, platelet counts naturally decline with age, a factor that may contribute to the heightened platelet reactivity observed in older populations [19,20,21]. Recent investigations have begun to elucidate the relationship between platelet function and AD, particularly focusing on the influence of platelets on cerebral blood flow and cognitive decline [22]. These findings underscore the multifunctional nature of platelets and their critical role in maintaining systemic health across different physiological and pathological contexts.

Compound K (CK), a primary metabolite of ginsenosides from *Panax ginseng*, exhibits superior bioavailability compared to its precursor [23,24,25,26,27,28,29]. CK has been shown to possess diverse pharmacological properties, including anti-diabetic, anti-carcinogenic, anti-inflammatory, anti-aging, and hepatoprotective effects, positioning it as a promising candidate for therapeutic applications [30,31,32,33]. In this study, we explored the effects of CK on apoptosis, differentiation, and the associated molecular mechanisms in chronic myeloid leukemia (CML) K562 and Meg-01 cell lines. Our results demonstrate that CK effectively inhibits cell growth and induces megakaryocytic differentiation in both cell lines. Additionally, we propose that the activation of the NLRP3 inflammasome pathway is a key mechanism underlying CK-mediated megakaryocytic differentiation, suggesting its potential as a therapeutic agent for enhancing platelet production.

## 2. Materials and Methods

### 2.1. Materials

Study materials included ginsenoside compound K (CK; ChemFaces, Wuhan, China), RPMI 1640 medium (Gibco, Grand Island, NY, USA), FBS (Gibco), penicillin–streptomycin (Sigma-Aldrich, St. Louis, MO, USA), 2′,7′-Dichlorofluorescein Di-acetate (H2DCFDA) (Invitrogen, Carlsbad, CA, USA), dihydroergotamine 123 (DHR 123) (Thermo Fisher Scientific, Waltham, MA, USA), diethylenetriaminepentaacetic acid (Sigma-Aldrich), D-Penicillamine (Sigma-Aldrich), dimethyl sulfoxide (DMSO) (Sigma-Aldrich), Ez-Cytox (DoGenBio, Seoul, Republic of Korea), Hoechst 33258, pentahydrate (Invitrogen), FITC Annexin V/Dead Cell Apoptosis Kit (Invitrogen), Total RNA Extraction Kit (SJ bio science, Incheon, Republic of Korea), Compact cDNA Synthesis Kit (SJ bio science), and SYBR Green Master Mix (SJ bio science).

### 2.2. Cell Culture and Treatment

The K562 cells were obtained from Korea Cell Line Bank (Seoul, Republic of Korea). The Meg-01 cells were kindly provided by Dr. Hyung Soo Kim (Department of Pharmaceutical Science and Technology, Kyungsung University, Busan, Republic of Korea). Both cell lines were cultured in RPMI 1640 medium supplemented with 10% fetal bovine serum (FBS) and 1% penicillin–streptomycin. Cultures were maintained at 37 °C in a humidified incubator with 5% CO_2_. For subculturing, cells and medium from a 100 × 10 mm culture dish were collected and centrifuged at 3000 rpm for 10 min. The supernatant was discarded, and the cell pellet was resuspended in 1 mL of fresh medium. The resuspended cells were then transferred to a new 100 × 10 mm culture dish containing 20 mL of medium. CK was dissolved in dimethyl sulfoxide (DMSO) to a concentration of 100 mM and stored at −20 °C until use. For drug treatment, CK and cells were diluted to twice the desired final concentration in a culture medium and then combined at a 1:1 volume ratio (*v*/*v*) to ensure simultaneous seeding and treatment. This approach was chosen to minimize the risk of cell damage due to high chemical concentrations, particularly in the suspension cultures.

### 2.3. Measurement of Cell Viability

Cell viability was assessed using the EZ-Cytox Kit, which employs a water-soluble tetrazolium salt (WST) for the MTT assay. Cells were seeded at a density of 2 × 10^5^ cells/well in a 96-well plate and simultaneously treated with CK for 48 and 72 h to determine cell viability and apoptosis. The cells were incubated at 37 °C in a humidified incubator with 5% CO_2_ throughout the treatment. Two hours before the completion of the treatment, 10 µL of EZ-Cytox reagent was added to each well containing the culture medium. Following the 2 h incubation with EZ-Cytox, absorbance was measured at 450 nm using an Epoch Microplate Spectrophotometer (BioTek, Winooski, VA, USA). Cell viability was calculated as a percentage relative to the vehicle control group.

### 2.4. Quantitative Real-Time PCR (q-PCR)

To evaluate the effect of CK on gene expression, K562 and Meg-01 cells were seeded at a density of 2.0 × 10^6^ cells per well in a 12-well plate and treated with either vehicle control or 3 and 5 µM of CK for 72 h. Total RNA was extracted using the Total RNA Extraction Kit according to the manufacturer’s instructions. The concentration of RNA was quantified, and cDNA synthesis was performed using a Compact cDNA Synthesis Kit. Quantitative real-time PCR (qPCR) was conducted in a total reaction volume of 20 µL, comprising 2 µL of cDNA, 10 µL of SYBR Green Master Mix, 6 µL of UltraPure™ DNase/RNase-Free Distilled Water (Invitrogen), and 1 µL each of forward and reverse primers. The thermal cycling conditions were as follows: an initial step at 50 °C for 2 min, followed by 10 min at 95 °C, then 40 cycles of denaturation at 95 °C for 15 s and annealing at 60 °C for 1 min. qPCR reactions were carried out using the QuantStudio 1 system (Applied Biosystems, Thermo Fisher Scientific) and analyzed with QuantStudio™ Design & Analysis Software v1.5.1 (Thermo Fisher Scientific). All gene expression levels were normalized to 18S ribosomal RNA using the comparative Ct method (2^−ΔΔCt^). The primer sequences used in this study are provided in Table 1.

### 2.5. Enzyme-Linked Immunosorbent Assay (ELISA)

The concentration of IL-1β was quantified using a quantitative sandwich enzyme immunoassay technique with the Human IL-1β ELISA Kit (Abclonal, Woburn, MA, USA). K562 and Meg-01 cells were cultured in a 48-well plate and divided into three groups: non-treated cells (vehicle control) and cells treated with 3 µM or 5 µM of CK. The cells were incubated at 37 °C for 72 h. After incubation, the culture samples were centrifuged at 3000 rpm for 10 min, and the supernatants were collected for analysis. Next, the samples and standards were added to the wells of microplates pre-coated with antibodies specific to human IL-1β. The plates were incubated at 37 °C for 2 h, followed by three washes. Subsequent incubation and washing steps for the enzyme conjugate were performed in accordance with the manufacturer’s protocol. After completing the assay, 50 µL of stop solution was added to each well, and absorbance was measured using a microplate reader. IL-1β concentrations were calculated based on the standard curve generated during the experiment.

### 2.6. Western Blot Analysis

To investigate cell differentiation and the mechanisms of apoptosis, Western blot analysis was conducted using specific antibodies against CD61, NLRP3, and caspase-1. K562 and Meg-01 cells were seeded at a density of 6.0 × 10^5^ cells per well in 6-well plates, treated with vehicle control or 3 µM and 5 µM of CK, and incubated for 72 h. Following incubation, cells were lysed with 200 µL of ProEXTM CETi Lysis Buffer containing Protease and Phosphatase Inhibitors and incubated on ice for 30 min. Lysates were centrifuged at 13,000 rpm for 10 min, and the supernatant was collected. Protein concentrations were determined using the Protein Assay Dye Reagent Concentrate. Cell lysates were prepared with 35 µg of protein in H_2_O and 5X SDS-PAGE Sample Buffer (TransLab). Equal amounts of protein were separated on 6–10% SDS-PAGE gels and transferred to polyvinylidene difluoride (PVDF) membranes (Millipore, Carrigtwohill, Ireland) by electrophoresis. Membranes were blocked using a Protein-Free Block Solution and subsequently incubated overnight at 4 °C with specific primary antibodies (1:1000 dilution) on a shaker. The following day, membranes were washed three times with TBS-T and incubated with IRDye 680RD Goat anti-Rabbit secondary antibody (1:10,000 dilution) (LI-COR, Lincoln, NE, USA) for 1 h and 30 min at room temperature on a shaker. After washing the membranes three times with TBS-T, protein bands were detected using the Odyssey Imaging System (Odyssey-XF, LI-COR). Protein band intensities were quantified using Empiria Studio software (version 2.3, LI-COR), with β-actin serving as the loading control. Original Western blot images can be found in Supplementary File S1.

### 2.7. Analysis of Cell Morphology

Hoechst 33258 staining was employed to observe morphological changes in K562 and Meg-01 cells. Cells were seeded at a density of 2.0 × 10^5^ cells/mL in 35 × 10 mm culture dishes, treated with vehicle control or 5 µM of CK, and incubated for 72 h. Following incubation, cells were stained with 5 µg/mL Hoechst 33258 and incubated for 20 min in the dark at 37 °C. After staining, cells were washed with PBS. Fluorescence microscopy was conducted using an Axio Observer microscope (Zeiss, Oberkochen, Germany) equipped with an LD Plan-NEOFLUAR 40X/0.6 Corr Ph2 objective. Image acquisition and analysis were performed using ZEN 3.6 (blue edition) microscopy software.

### 2.8. Flow Cytometric Analysis

The FITC Annexin V/Dead Cell Apoptosis Kit was employed to measure apoptosis in K562 and Meg-01 cells, following the manufacturer’s instructions. Cells were seeded in 35 × 10 mm culture dishes and treated with vehicle control or CK at concentrations of 3 µM and 5 µM for 72 h. After treatment, cells were collected at a density of 1 × 10^6^ cells/mL and washed with ice-cold PBS. The cells were then resuspended in a buffer containing propidium iodide (PI), Annexin V, and FITC and incubated at room temperature for 15 min. Following incubation, the cells were treated with 1X annexin-binding buffer. Apoptotic cells were subsequently analyzed using CytoFLEX flow cytometry (Beckman Coulter, Brea, CA, USA). For K562 cells, up to 30,000 events per sample were recorded, while up to 10,000 events per sample were detected during flow cytometric analysis of Meg-01 cells.

### 2.9. RT^2^ Profiler PCR Arrays

A PCR array was conducted to identify apoptosis-related pathways. RNA samples were prepared following the same protocol as described in the qPCR section above. For the assay, vehicle control (Con) and 5 µM CK-treated samples were used, excluding the 3 µM CK condition. After quantification of the extracted mRNA, cDNA was synthesized using the RT^2^ First Strand Kit. PCR reactions were assembled by adding RT^2^ SYBR Green Master Mix, cDNA, and UltraPure™ DNase/RNase-Free Distilled Water to each well, following the manufacturer’s instructions. Quantitative PCR (qPCR) arrays were performed using the RT^2^ Profiler PCR arrays, which included 84 apoptosis-related genes, 5 housekeeping genes, and 3 control genes. The reverse transcription reactions were carried out using the QuantStudio 1 system (Applied Biosystems) under the same cycling conditions described previously. The resulting threshold cycle (Ct) value was uploaded to the web-based GeneGlobe Data Analysis Centre, and 2^−ΔΔCt^ was used to calculate relative gene expression. The list of genes used in the experiment is provided in Table S1.

### 2.10. Statistical Analysis

Statistical analysis of the experimental data was performed using GraphPad Prism version 5.0 (GraphPad Software, Inc., San Diego, CA, USA). A one-way analysis of variance (ANOVA) followed by Bonferroni and Dunnett’s multiple comparison tests was used to assess differences between groups. A *p*-value of less than 0.05 (*p* < 0.05) was considered statistically significant. Data are presented as the mean ± standard error of the mean (SEM).

## 3. Results

### 3.1. Measurement of Cell Viability of K562 Cells and Meg-01 Cells

**Figure 1:**
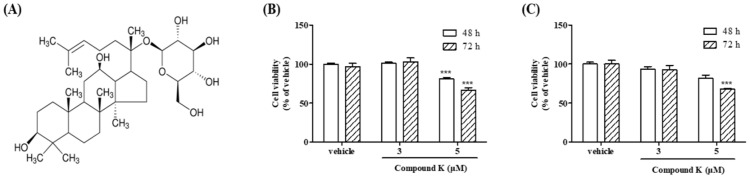
Effects of compound K (CK) on the cell growth of K562 and Meg01 cells. (**A**) The chemical structure of CK. Cell viability was measured using an MTT assay. (**B**) K562 and (**C**) Meg-01 cells were treated with vehicle (0.1% DMSO) or CK (3 or 5 µM) for 48 h or 72 h. One-way ANOVA with Bonferroni test was used to determine the significance of differences: *** *p* < 0.001 compared to the vehicle-treated group.

K562 and Meg-01 cell lines are well-established models of human CML [34,35]. To determine the optimal experimental concentrations of CK, we assessed its effects on the viability of these CML cell lines (Figure 1). Cells were treated with the vehicle control or CK at concentrations of 3 µM and 5 µM for 48 h and 72 h, and cell viability was measured using the MTT assay. As shown in Figure 1B, CK treatment significantly decreased the viability of K562 cells. After 48 h of treatment, cell viability decreased by 18.89% compared to the control for 5 µM CK. Following 72 h of treatment, cell viability further declined by 31.11% compared to the control (*** *p* < 0.001). Similarly, in Meg-01 cells (Figure 1C), CK treatment at 3 µM and 5 µM for 48 h reduced viability by 6.50% and 18.17% compared to the control, respectively. After 72 h of treatment, cell viability decreased further by 7.28% and 32.25% compared to the control, respectively (*** *p* < 0.001). Also, as shown in Figure 1C, the viability of Meg-01 cells treated with CK (3 and 5 µM) for 48 h decreased by 6.50% and 18.17% compared to the vehicle control, respectively, while the viability of cells treated for 72 h decreased by 7.28% and 32.25% compared to the control, respectively (*** *p* < 0.001). These findings demonstrate that CK significantly inhibits the viability of both K562 and Meg-01 cells in a dose- and time-dependent manner, with 5 µM CK exhibiting a pronounced inhibitory effect on cell growth.

### 3.2. CK Promoted Megakaryocytic Differentiation of K562 and Meg-01 Cells

**Figure 2:**
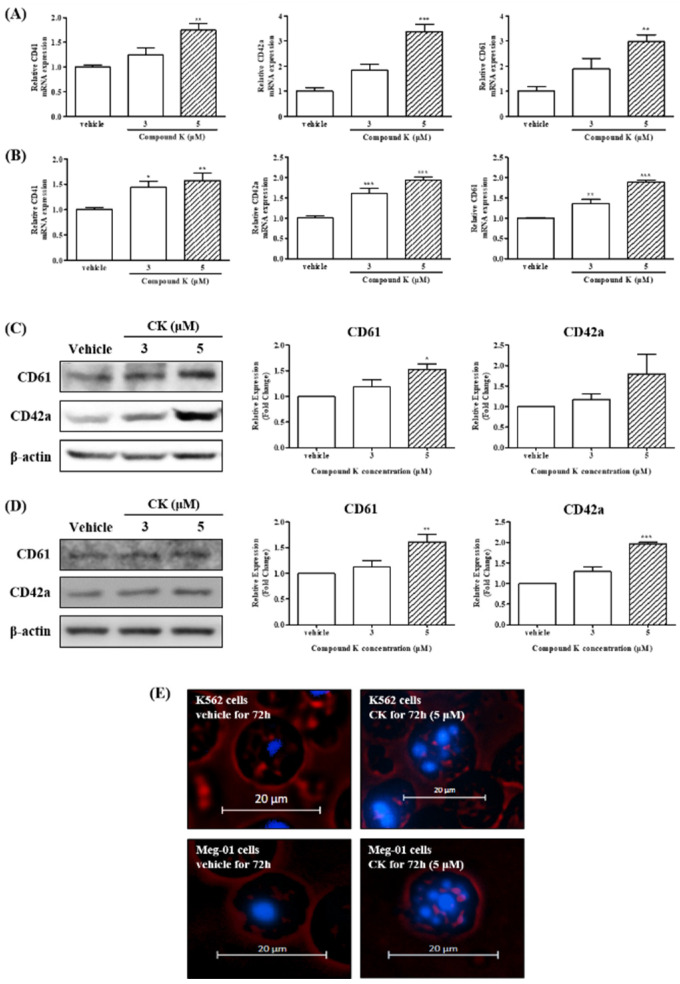
Effects of CK on the expression of megakaryocytic differentiation genes in K562 and Meg-01 cells. K562 and Meg-01 cells were treated with vehicle or CK (3 or 5 µM) for 72 h. The messenger RNA (mRNA) expression levels in (**A**) K562 and (**B**) Meg-01 cells. CK induced the secretion of differentiation markers. The protein expression of CD61, CD42a, and β-actin was measured by Western blot analysis (*n* = 3). Quantification results of (**C**) K562 and (**D**) Meg-01 cells and a representative blot are shown. One-way ANOVA with Bonferroni test was used to determine the significance of differences: * *p* < 0.05, ** *p* < 0.01, and *** *p* < 0.001 compared to vehicle-treated cells. (**E**) Morphological changes of the cells and multi-lobulation of the nucleus were observed under microscope. K562 and Meg-01 cells were treated with vehicle (0.1% DMSO) or 5 µM CK for 72 h. Representative histograms are shown. Blue fluorescence indicates nuclear staining by Hoechst. Red indicates a cell membrane that has been unstained. The scale bar is 20 µm.

K562 and Meg-01 cells are widely used models for investigating hematopoietic differentiation, as they can be induced to differentiate into various hematopoietic lineages with appropriate agents [34,36]. In this study, we examined whether CK could induce megakaryocytic differentiation in K562 and Meg-01 cells. As shown in Figure 2, CK treatment (5 µM) significantly upregulated the mRNA expression of megakaryocytic differentiation markers, including CD41, CD42a, and CD61, in K562 cells after 72 h of treatment (** *p* < 0.01 and *** *p* < 0.001) (Figure 2A). Similarly, in Meg-01 cells, CK treatment at both 3 µM and 5 µM for 72 h significantly increased the mRNA expression of CD41, CD42a, and CD61 (* *p* < 0.05, ** *p* < 0.01, and *** *p* < 0.001) (Figure 2B). Consistent with the gene expression results, in K562 cells treated with 5 µM CK for 72 h, protein expression of CD61 and CD42a was significantly increased to 52.63% and 79.93%, respectively, compared to the vehicle-treated group (* *p* < 0.05). Meg-01 cells were also significantly increased at 61.33% and 96.79% compared to the vehicle-treated group (* *p* < 0.05, ** *p* < 0.01, and *** *p* < 0.001) (Table 2 and Figure 2C,D). These results indicate that CK markedly promotes the expression of CD61, a crucial megakaryocytic marker, at both the gene and protein levels in K562 and Meg-01 cells.

### 3.3. CK Promoted Multi-Lobulated Nuclei in K562 and Meg-01 Cells

MKs are known to undergo characteristic morphological changes during differentiation and maturation, including the formation of multi-lobulated nuclei. To assess whether CK induces such morphological changes, we examined nuclear multi-lobulation in K562 and Meg-01 cells using Hoechst 33258 staining and fluorescence microscopy. As shown in Figure 2E, treatment with 0.1% DMSO (vehicle control) had no significant effect, whereas treatment with CK (5 µM) notably promoted the formation of multi-lobulated nuclei in both K562 and Meg-01 cells. These findings suggest that CK enhances megakaryocytic differentiation in CML K562 and Meg-01 cells by promoting nuclear multi-lobulation, a hallmark of MK maturation.

### 3.4. Expression of Egr-1 Induced by CK Could Promote Megakaryocytic Differentiation of CML Cells

**Figure 3:**
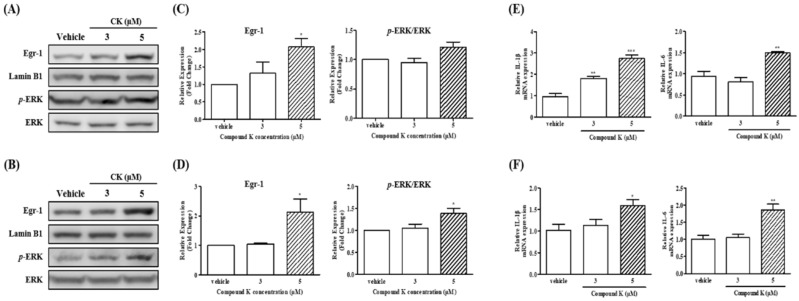
Involvement of CK in the megakaryocytic differentiation-related signaling pathway-dependent gene expression activation in CML cells. Cells were treated with vehicle or CK (3 or 5 µM) for 72 h. Western blot analysis measured the levels of Egr-1, Lamin B1, phosphor-ERK (p-ERK), and ERK protein. A representative blot of (**A**) K562 and (**B**) Meg-01 cells is shown. The experiments were performed in triplicate, and the data represent the mean ± SD of independent experiments of (**C**) K562 and (**D**) Meg-01 cells. The mRNA expression levels in (**E**) K562 and (**F**) Meg-01 cells. One-way ANOVA with Bonferroni test was used to determine the significance of differences: * *p* < 0.05, ** *p* < 0.01, and *** *p* < 0.001 compared to vehicle-treated cells.

Early growth response 1 (Egr-1) is a multifunctional transcription factor that regulates various cellular processes, including cell growth, differentiation, and apoptosis, in response to cytokine signaling and growth factors. To investigate whether the Egr-1 gene is involved in CK-induced megakaryocytic differentiation, we assessed its protein levels following CK treatment. As shown in Figure 3, treatment with CK (5 µM) for 72 h significantly increased Egr-1 protein levels in both K562 and Meg-01 cells (* *p* < 0.05). The induction of Egr-1 is known to be mediated by extracellular signal-regulated kinase (ERK). To further confirm CK-induced Egr-1 activation, we measured ERK protein levels under the same experimental conditions. As shown in Figure 3D, CK (5 µM) treatment significantly increased ERK levels in Meg-01 cells (* *p* < 0.05), consistent with the induction of Egr-1. These results suggest that CK-induced Egr-1 expression may be mediated through ERK activation in CML cells, promoting megakaryocytic differentiation.

### 3.5. Effects of CK on mRNA Expression of Megakaryocytic Differentiation-Related Cytokines in K562 and Meg-01 Cells

The proliferation and maturation of MK precursors are indirectly regulated by various cytokines, including interleukin (IL)-1β, IL-3, and IL-6. These cytokines stimulate TPO, which in turn promotes the differentiation and maturation of MK precursors, ultimately leading to platelet production [37]. In this study, we examined the effect of CK on the expression of IL-1β and IL-6, two key cytokines known to stimulate TPO, in K562 and Meg-01 cells. As shown in Figure 3E,F, treatment with 5 µM CK significantly increased the mRNA expression of both IL-1β and IL-6 in K562 and Meg-01 cells (* *p* < 0.05, ** *p* < 0.01, and *** *p* < 0.001). These findings suggest that CK may enhance TPO production by upregulating specific cytokines, thereby promoting the differentiation and maturation of MK precursors in these cell lines.

### 3.6. Effects of CK on Apoptosis of K562 and Meg-01 Cells

**Figure 4:**
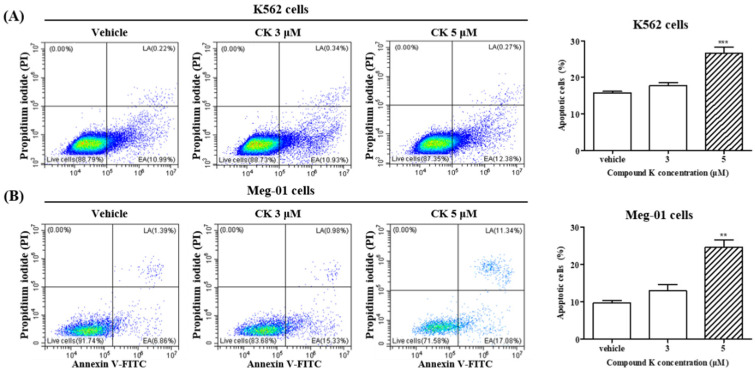
Effect of CK on apoptosis of K562 cells and Meg-01 cells. The population of early apoptotic (EA) and late apoptotic (LA) cells was detected using an Annexin V-FITC and propidium iodide (PI) staining kit, according to the manufacturer’s instructions. A representative histogram is shown for (**A**) K562 and (**B**) Meg-01 cells. The apoptotic cell populations in EA and LA phases were quantified. The experiments were replicated three times, and the data represent the mean ± SEM of independent experiments. One-way ANOVA with Bonferroni test was used to determine the significance of differences: ** *p* < 0.01 and *** *p* < 0.001 compared to vehicle-treated cells.

It is widely accepted that apoptosis is essential for platelet biogenesis [38,39,40,41]. Recently, it was observed that MKs and platelets depend on apoptosis for their development and viability [42]. We investigated the effect of CK on apoptosis in K562 and Meg-01 cells. Cells were treated with the vehicle control or CK at concentrations of 3 µM and 5 µM for 72 h, and apoptosis was assessed via flow cytometry. As depicted in Table 2 and Figure 4, CK treatment significantly increased apoptosis in both cell lines. In K562 cells, apoptosis increased to 17.71% with 3 µM CK and 26.59% with 5 µM CK. Similarly, in Meg-01 cells, apoptosis levels rose to 13.01% with 3 µM CK and 24.62% with 5 µM CK. Furthermore, the proportion of early apoptotic and late apoptotic cells was significantly elevated in the 5 µM CK-treated group compared to the vehicle-treated control (** *p* < 0.01 and *** *p* < 0.001). Apoptosis shares several features with the platelet assembly process in MKs, including cytoskeletal reorganization. These findings suggest that CK induces apoptosis, an essential component of platelet biogenesis, in K562 and Meg-01 cells.

### 3.7. CK Activates NLRP3 Inflammasome in K562 and Meg-01 Cells

**Figure 5:**
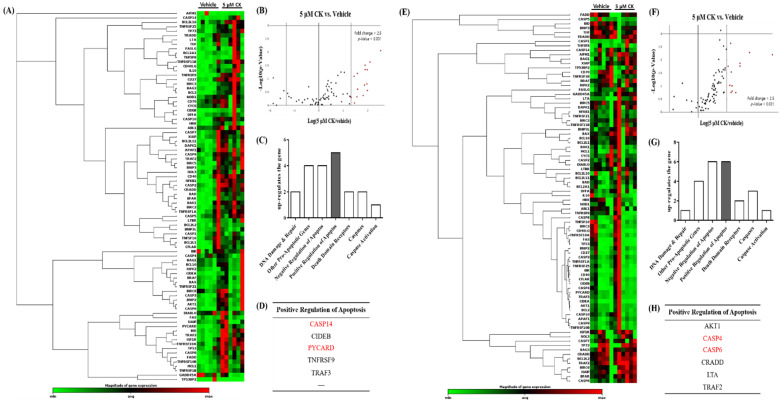
Summary of the RT^2^ Profiler PCR array analysis of the apoptosis pathway. The hierarchical clustering of gene signatures was determined using PCR arrays in (**A**) K562 cells and (**E**) Meg-01 cells. (**B**,**F**) The volcano plots identify significant changes in gene expression between groups. Cluster analysis revealed that CK upregulates the genes in (**C**) K562 and (**G**) Meg-01 cells in the positive regulation signaling pathway. (**D**,**H**) List of positive regulation-related genes regulated by CK. There is a distinct increase in the inflammasome expression among the positive regulation-related genes. Red indicates upregulation of inflammasome genes (at least 2.5-fold).

Apoptosis can be triggered through various pathways, including the NLRP3 inflammasome, endoplasmic reticulum (ER) stress, and mitochondrial dysfunction. To elucidate the molecular mechanisms underlying CK-induced apoptosis, we conducted pathway-focused gene expression profiling using the RT^2^ Profiler PCR Array System, which targets 84 genes associated with apoptosis in K562 and Meg-01 cells. Differentially expressed genes were identified based on a fold change greater than 2.5 (*p* < 0.001). K562 and Meg-01 cells treated with 5 µM CK exhibited distinct gene expression profiles compared to vehicle-treated controls. As shown in Table S2 and Figure 5B, twelve genes were significantly upregulated (>2.5-fold) in K562 cells following CK treatment, including CASP14, CIDEB, PYCARD, TNFRSF9, and TRAF3, all of which are involved in the positive regulation of apoptosis. Additionally, anti-apoptotic genes such as BCL2, BIRC3, CD40LG, and CIDEA were upregulated. In Meg-01 cells, 16 genes were upregulated by more than 2.5-fold after CK exposure, including AKT1, CASP4, CASP6, CRADD, LTA, TRAF2, BAG3, BCL2L2, BFAR, BIRC2, IGF1R, and XIAP (Table S3 and Figure 5F). When comparing the upregulated apoptosis-related genes in K562 and Meg-01 cells, the positive regulation of the apoptosis pathway showed the highest number of upregulated genes, with five genes in K562 cells and six genes in Meg-01 cells (Figure 5C,G). Notably, four of these genes—CASP4, CASP6, CASP14, and PYCARD—are inflammasome-related (Figure 5D,H). These findings underscore the involvement of the inflammasome pathway, particularly the NLRP3 inflammasome, in CK-induced apoptosis, warranting further investigation.

### 3.8. NLRP3 Inflammasome Activation Induced by CK Could Promote Apoptosis of K562 and Meg-01 Cells

**Figure 6:**
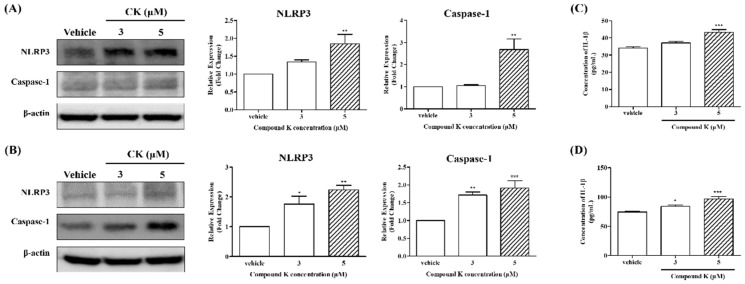
Effects of CK on the expression of the complexes of NLRP3 inflammatory markers in K562 and Meg-01 cells. Human chronic myeloid leukemia (CML) cells were treated with vehicle or CK (3 or 5 µM) for 72 h. The protein expression of NLRP3, caspase-1, and actin was measured by Western blot analysis (*n* = 3). Quantification results of (**A**) K562 and (**B**) Meg-01 cells and a representative blot is shown. Also shown are the ELISA results for inflammatory cytokine IL-1ß in the vehicle or CK (3 or 5 µM) groups. IL-1β concentration was significantly increased by CK treatment in (**C**) K562 and (**D**) Meg-01 cells. The mean ± SD of three independent experiments is also shown. One-way ANOVA with Bonferroni test was used to determine the significance of differences: * *p* < 0.05, ** *p* < 0.01, and *** *p* < 0.001 compared to vehicle-treated cells.

To further investigate the involvement of the inflammasome pathway in CK-induced apoptosis, we conducted Western blot analysis to assess the protein expression of key components of the NLRP3 inflammasome complex, including NLRP3 and caspase-1, in K562 and Meg-01 cells treated with 3 µM and 5 µM CK. As shown in Figure 6A,B, treatment with 5 µM CK led to significant increases in the protein expression of both NLRP3 and caspase-1 in K562 and Meg-01 cells (** *p* < 0.01 and *** *p* < 0.001). These findings suggest that CK may activate the NLRP3 inflammasome complex, potentially promoting platelet production by inducing apoptosis. Activation of the inflammasome is known to trigger the production of pro-inflammatory cytokines, such as IL-1β, and initiate apoptotic pathways [43]. To further confirm NLRP3 inflammasome activation, we measured IL-1β levels by ELISA under the same experimental conditions as previous studies. The results demonstrate a significant increase in IL-1β levels in the 5 µM CK-treated group compared to the vehicle group (*** *p* < 0.001).

## 4. Discussion

Platelets, produced by MKs derived from bone marrow cells, are essential for maintaining hemostasis by facilitating blood clotting at injury sites [1,44]. Dysfunction in platelet production, characterized by a reduction in platelet count, can result from various underlying factors and is associated with several diseases, including leukemia, hepatitis, and AD [17,18]. Enhancing megakaryocytic differentiation is a critical process for augmenting the production of healthy platelets [13]. Recent studies have focused on strategies to improve megakaryocytic differentiation, which could lead to increased platelet production and offer potential therapeutic approaches for platelet-related disorders [45].

While platelets are crucial for hemostasis, they can be detrimental in certain conditions, such as cancer, where they promote metastasis [46]. In such cases, reducing platelet activation or count is the desired therapeutic approach. However, our study focuses on the potential therapeutic effects of CK in conditions where enhancing platelet production is beneficial. For instance, in thrombocytopenia or other platelet-related disorders characterized by abnormally low platelet counts, increasing platelet production is essential [47]. Additionally, recent research has suggested that reduced platelet counts may be associated with neurodegenerative diseases, including Alzheimer’s disease [48].

Megakaryocytic differentiation is a complex, multi-step process characterized by the expression of MK-specific markers and the generation of multi-lobulated, polyploid cells through endomitosis. In this study, we explored the effects of CK on the induction of megakaryocytic differentiation and its potential underlying molecular mechanisms in K562 and Meg-01 cells. Our findings demonstrate that CK significantly promotes the expression of megakaryocytic differentiation markers (Figure 2) and facilitates multi-lobulated nucleus formation during megakaryocytic differentiation (Figure 2E), suggesting its capacity to enhance megakaryocyte maturation.

Thrombopoietin (TPO) is the primary hormone responsible for driving MK development [6,9]. TPO stimulates HSCs, thereby promoting the growth of MK colonies and facilitating the proliferation and differentiation of MK progenitors [11]. Pro-inflammatory cytokines such as IL-1β and IL-6, regulated by EGR1, have been identified as key enhancers of TPO production [12]. Notably, previous studies have demonstrated that EGR1 activation is linked to ERK signaling [49]. In line with these findings, our study shows that CK significantly stimulates the production of megakaryocytic differentiation-associated cytokines, particularly IL-1β and IL-6, via ERK-induced EGR1 activation in K562 and Meg-01 cells (Figure 3). Importantly, this study is the first to demonstrate that CK promotes megakaryocytic differentiation and elucidates the underlying molecular mechanisms, specifically through the activation of the ERK/EGR1 signaling pathway.

Recent studies have suggested that apoptosis is the final physiological fate of mature MKs [28,50,51,52,53,54]. In alignment with the induction of megakaryocytic differentiation, our findings indicate that CK-induced megakaryocytic differentiation is accompanied by an increase in apoptosis in K562 and Meg-01 cells (Figure 4). The increased apoptosis observed during CK-induced differentiation suggests that CK may facilitate the maturation and development of MKs by activating the apoptotic pathway, thereby promoting the production of functional platelets. The summary of apoptosis and megakaryocyte differentiation gene expression is included in Table 2.

Using RT^2^ Profiler PCR array analysis, we observed a significant upregulation of genes associated with the NLRP3 inflammasome, indicating that CK may induce apoptosis through the activation of this inflammasome pathway (Figure 5). These findings are consistent with previous studies that have suggested a link between CK and the activation of the NLRP3 inflammasome. The NLRP3 inflammasome has been implicated in various conditions, including atherosclerosis, neurodegenerative diseases, and autoimmune disorders [43,55,56]. Interestingly, CK has also been reported to suppress NLRP3 inflammasome activation, which may have important therapeutic implications in mitigating inflammatory responses associated with these diseases [56]. While many studies emphasize the suppressive effects of CK on NLRP3 inflammasome activation, it is important to note that NLRP3 inflammasome activation can also induce megakaryocytic differentiation. These seemingly contrasting findings may be attributable to differences in cell types or specific experimental conditions, warranting further investigation into the precise role of the NLRP3 inflammasome in CK-induced megakaryocytic differentiation.

**Figure A1:**
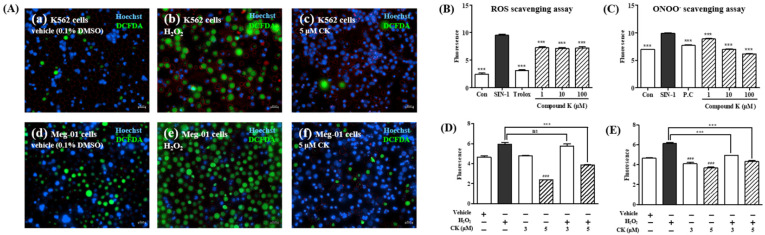
Effects of CK on reactive oxygen species (ROS) levels. (**A**) Intracellular ROS detection was performed using fluorescence microscopy. Intracellular ROS was measured by DCFDA staining after (**a**,**d**) vehicle, (**b**,**e**) H_2_O_2_, or (**c**,**f**) 5 μM CK treatment. Representative fluorescence images of (**a**–**c**) K562 and (**d**–**f**) Meg-01 cells. The intensity of the green fluorescence indicates ROS concentration in the cells. (**B**) ROS scavenging assay after CK exposure. The effect of CK on ROS production was evaluated. ROS levels were quantitatively determined using the H_2_DCFDA (*n* = 4). Trolox was used as the positive control (P.C). (**C**) The effect of CK on peroxynitrite (ONOO^−^) production was evaluated (*n* = 4). Penicillamine was used as the P.C. The scavenging activity of CK on ROS and ONOO^−^ induced by 10 µM SIN-1 was estimated in a cell-free in vitro culture. One-way ANOVA with Bonferroni test was used to determine the significance of differences: *** *p* < 0.001 compared to SIN-1-treated cells. The scavenging activity of CK on ROS induced by H_2_O_2_ was estimated in (**D**) K562 and (**E**) Meg-01 cells. One-way ANOVA with Bonferroni test was used to determine the significance of differences: *** *p* < 0.001 compared to H_2_O_2_-treated cells. ### *p* < 0.001 compared to vehicle-treated cells. “ns” represents no significant differences compared to H_2_O_2_-treated cells.

Moreover, our results demonstrate that CK-induced megakaryocytic differentiation is mediated through the activation of the NLRP3 inflammasome, a key molecular complex involved in regulating inflammatory responses (Figure 6). Notably, CK-induced activation of the NLRP3 inflammasome also triggered apoptosis, independent of reactive oxygen species (ROS) production (Figure A1). These findings suggest that CK may enhance platelet production and improve platelet function, positioning it as a promising candidate for the therapeutic management of platelet-related disorders and other associated diseases. CK is a major bioactive component of Panax ginseng, belonging to the dammarane-type saponins. As a metabolite of protopanaxadiol (PPD), CK exhibits superior bioavailability compared to other ginsenosides and has demonstrated various pharmacological properties, including anti-diabetic, anti-cancer, and anti-aging effects [23,24,29,30,31,32,33]. Traditionally, differentiation-inducing substances have been associated with the induction of apoptosis via ROS generation [57]. Interestingly, our findings revealed that CK (5 µM) inhibited ROS levels without inducing cell death. These observations suggest that CK differs mechanistically from other differentiation-inducing agents, indicating a potential novel pathway for its effects. Further studies are warranted to elucidate the precise mechanisms by which CK influences differentiation and cell survival.

**Figure 7:**
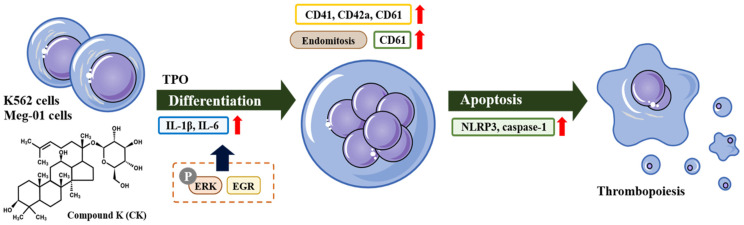
Summary of the experiments on the effect of CK on megakaryocytic differentiation in CML K562 and Meg-01 cells. The observation of specific expression markers in megakaryocytic differentiation and cell morphology confirmed CK’s facilitation of differentiation. Additionally, CK reduces cell viability and promotes megakaryocytic differentiation through the activation of the NLRP3 inflammasome in human CML K562 and Meg-01 cells.

In conclusion, this study demonstrates that CK induces the upregulation of genes associated with the NLRP3 inflammasome, suggesting its activation (Figure 7). These findings are consistent with previous research and enhance our understanding of CK’s role in modulating inflammatory responses. The potential therapeutic implications of CK-mediated NLRP3 inflammasome activation merit further investigation, particularly in inflammation-related diseases, to fully elucidate its broader clinical applications. While our study focuses on the potential effects of CK in promoting megakaryocytic differentiation and apoptosis, it is important to note that limited research has been conducted on the potential side effects or toxicity of CK in humans or other cell types. Further studies are needed to evaluate the long-term safety and possible adverse effects of CK and ensure its suitability for therapeutic agents.

## Figures and Tables

**Table 1:** Primer sequences used for q-PCR analysis.

	Primer Sequence (5′→3′)		
Gene	Forward Primer	Reverse Primer	Scale
18S	5′-CTCTAGATAACCTCGGGCCG-3′	5′-GTCGGGAGTGGGTAATTTGC-3′	25 nmole
CD41	5′-GATGAGACCCGAAATGTAGGC-3′	5′-GTCTTTTCTAGGACGTTCCAGTG-3′	25 nmole
CD42a	5′-ACCCTCGATGTGACGCAGA-3′	5′-CCAGAGGCGCAGATAGGTG-3′	25 nmole
CD61	5′-GTGACCTGAAGGAGAATCTGC-3′	5′-CCGGAGTGCAATCCTCTGG-3′	25 nmole
IL-1ß	5′-ATGATGGCTTATTACAGTGGCAA-3′	5′-GTCGGAGATTCGTAGCTGGA-3′	25 nmole
IL-6	5′-ACTCACCTCTTCAGAACGAATTG-3′	5′-CCATCTTTGGAAGGTTCAGGTTG-3′	25 nmole

**Table 2:** Summary of apoptosis and megakaryocyte differentiation gene expression in CML.

Compared to the Vehicle-Treated Group		K562		Meg-01	
		3 µM	5 µM	3 µM	5 µM
The protein expression level of	CD61 (%)	18.58%	52.63%	12.02%	61.33%
	CD42a (%)	17.54%	79.93%	29.33%	96.79%
Cells with apoptosis (%)		17.71%	26.59%	13.01%	24.62%

## Data Availability

Data are available upon request.

## Supplementary Materials

The following supporting information can be downloaded at: https://www.mdpi.com/article/10.3390/biom14101257/s1, Table S1: Genes used for RT2 Profiler PCR array analysis; Table S2: Summarizes the effects of CK on apoptosis-related gene expression in K562 cells; Table S3: Summarizes the effects of CK on apoptosis-related gene expression in Meg-01 cells. Original Western blot images can be found in Supplementary File S1.
