# Supplementary material for: Compound K Promotes Megakaryocytic Differentiation by NLRP3 Inflammasome Activation
Source: Biomolecules. 2024 Oct 4;14(10):1257. doi: 10.3390/biom14101257 (PMC11506438; doi:10.3390/biom14101257)
Supplement: Supplementary file 1 [file biomolecules-14-01257-s001.zip › biomolecules-3208894-supplementary.pdf]

Table S1. Genes used for RT^2 Profiler PCR array analysis

POSITION	UNIGENE	REFSEQ	SYMBOL	DESCRIPTION
**A01**	Hs.431048	NM_005157	ABL1	C-abl oncogene 1, non-receptor tyrosine kinase
**A02**	Hs.424932	NM_004208	AIFM1	Apoptosis-inducing factor, mitochondrion-associated, 1
**A03**	Hs.525622	NM_005163	AKT1	V-akt murine thymoma viral oncogene homolog 1
**A04**	Hs.552567	NM_001160	APAF1	Apoptotic peptidase activating factor 1
**A05**	Hs.370254	NM_004322	BAD	BCL2-associated agonist of cell death
**A06**	Hs.377484	NM_004323	BAG1	BCL2-associated athanogene
**A07**	Hs.523309	NM_004281	BAG3	BCL2-associated athanogene 3
**A08**	Hs.485139	NM_001188	BAK1	BCL2-antagonist/killer 1
**A09**	Hs.624291	NM_004324	BAX	BCL2-associated X protein
**A10**	Hs.193516	NM_003921	BCL10	B-cell CLL/lymphoma 10
**A11**	Hs.150749	NM_000633	BCL2	B-cell CLL/lymphoma 2
**A12**	Hs.227817	NM_004049	BCL2A1	BCL2-related protein A1
**B01**	Hs.516966	NM_138578	BCL2L1	BCL2-like 1
**B02**	Hs.283672	NM_020396	BCL2L10	BCL2-like 10 (apoptosis facilitator)
**B03**	Hs.469658	NM_006538	BCL2L11	BCL2-like 11 (apoptosis facilitator)
**B04**	Hs.410026	NM_004050	BCL2L2	BCL2-like 2
**B05**	Hs.435556	NM_016561	BFAR	Bifunctional apoptosis regulator
**B06**	Hs.517145	NM_001196	BID	BH3 interacting domain death agonist
**B07**	Hs.475055	NM_001197	BIK	BCL2-interacting killer (apoptosis-inducing)
**B08**	Hs.696238	NM_001166	BIRC2	Baculoviral IAP repeat containing 2
**B09**	Hs.127799	NM_001165	BIRC3	Baculoviral IAP repeat containing 3
**B10**	Hs.744872	NM_001168	BIRC5	Baculoviral IAP repeat containing 5
**B11**	Hs.150107	NM_016252	BIRC6	Baculoviral IAP repeat containing 6
**B12**	Hs.646490	NM_004330	BNIP2	BCL2/adenovirus E1B 19kDa interacting protein 2
**C01**	Hs.144873	NM_004052	BNIP3	BCL2/adenovirus E1B 19kDa interacting protein 3
**C02**	Hs.131226	NM_004331	BNIP3L	BCL2/adenovirus E1B 19kDa interacting protein 3-like
**C03**	Hs.550061	NM_004333	BRAF	V-raf murine sarcoma viral oncogene homolog B1
**C04**	Hs.2490	NM_033292	CASP1	Caspase 1, apoptosis-related cysteine peptidase (interleukin 1, beta, convertase)
**C05**	Hs.5353	NM_001230	CASP10	Caspase 10, apoptosis-related cysteine peptidase
**C06**	Hs.466057	NM_012114	CASP14	Caspase 14, apoptosis-related cysteine peptidase
**C07**	Hs.368982	NM_032982	CASP2	Caspase 2, apoptosis-related cysteine peptidase
**C08**	Hs.141125	NM_004346	CASP3	Caspase 3, apoptosis-related cysteine peptidase
**C09**	Hs.138378	NM_001225	CASP4	Caspase 4, apoptosis-related cysteine peptidase
**C10**	Hs.213327	NM_004347	CASP5	Caspase 5, apoptosis-related cysteine peptidase
**C11**	Hs.654616	NM_032992	CASP6	Caspase 6, apoptosis-related cysteine peptidase
**C12**	Hs.9216	NM_001227	CASP7	Caspase 7, apoptosis-related cysteine peptidase
**D01**	Hs.599762	NM_001228	CASP8	Caspase 8, apoptosis-related cysteine peptidase
**D02**	Hs.329502	NM_001229	CASP9	Caspase 9, apoptosis-related cysteine peptidase
**D03**	Hs.355307	NM_001242	CD27	CD27 molecule
**D04**	Hs.472860	NM_001250	CD40	CD40 molecule, TNF receptor superfamily member 5
**D05**	Hs.592244	NM_000074	CD40LG	CD40 ligand
**D06**	Hs.501497	NM_001252	CD70	CD70 molecule

POSITION	Unigene	Refseq	Symbol	Description
**D07**	Hs.390736	NM_003879	CFLAR	CASP8 and FADD-like apoptosis regulator
**D08**	Hs.249129	NM_001279	CIDEA	Cell death-inducing DFFA-like effector a
**D09**	Hs.642693	NM_014430	CIDEB	Cell death-inducing DFFA-like effector b
**D10**	Hs.38533	NM_003805	CRADD	CASP2 and RIPK1 domain containing adaptor with death domain
**D11**	Hs.437060	NM_018947	CYCS	Cytochrome c, somatic
**D12**	Hs.380277	NM_004938	DAPK1	Death-associated protein kinase 1
**E01**	Hs.484782	NM_004401	DFFA	DNA fragmentation factor, 45kDa, alpha polypeptide
**E02**	Hs.169611	NM_019887	DIABLO	Diablo, IAP-binding mitochondrial protein
**E03**	Hs.86131	NM_003824	FADD	Fas (TNFRSF6)-associated via death domain
**E04**	Hs.667309	NM_000043	FAS	Fas (TNF receptor superfamily, member 6)
**E05**	Hs.2007	NM_000639	FASLG	Fas ligand (TNF superfamily, member 6)
**E06**	Hs.80409	NM_001924	GADD45A	Growth arrest and DNA-damage-inducible, alpha
**E07**	Hs.87247	NM_003806	HRK	Harakiri, BCL2 interacting protein (contains only BH3 domain)
**E08**	Hs.643120	NM_000875	IGF1R	Insulin-like growth factor 1 receptor
**E09**	Hs.193717	NM_000572	IL10	Interleukin 10
**E10**	Hs.36	NM_000595	LTA	Lymphotoxin alpha (TNF superfamily, member 1)
**E11**	Hs.1116	NM_002342	LTBR	Lymphotoxin beta receptor (TNFR superfamily, member 3)
**E12**	Hs.632486	NM_021960	MCL1	Myeloid cell leukemia sequence 1 (BCL2-related)
**F01**	Hs.646951	NM_004536	NAIP	NLR family, apoptosis inhibitory protein
**F02**	Hs.618430	NM_003998	NFKB1	Nuclear factor of kappa light polypeptide gene enhancer in B-cells 1
**F03**	Hs.738731	NM_006092	NOD1	Nucleotide-binding oligomerization domain containing 1
**F04**	Hs.513667	NM_003946	NOL3	Nucleolar protein 3 (apoptosis repressor with CARD domain)
**F05**	Hs.499094	NM_013258	PYCARD	PYD and CARD domain containing
**F06**	Hs.103755	NM_003821	RIPK2	Receptor-interacting serine-threonine kinase 2
**F07**	Hs.241570	NM_000594	TNF	Tumor necrosis factor
**F08**	Hs.591834	NM_003844	TNFRSF10A	Tumor necrosis factor receptor superfamily, member 10a
**F09**	Hs.661668	NM_003842	TNFRSF10B	Tumor necrosis factor receptor superfamily, member 10b
**F10**	Hs.81791	NM_002546	TNFRSF11B	Tumor necrosis factor receptor superfamily, member 11b
**F11**	Hs.713833	NM_001065	TNFRSF1A	Tumor necrosis factor receptor superfamily, member 1A
**F12**	Hs.256278	NM_001066	TNFRSF1B	Tumor necrosis factor receptor superfamily, member 1B
**G01**	Hs.443577	NM_014452	TNFRSF21	Tumor necrosis factor receptor superfamily, member 21
**G02**	Hs.462529	NM_003790	TNFRSF25	Tumor necrosis factor receptor superfamily, member 25
**G03**	Hs.738942	NM_001561	TNFRSF9	Tumor necrosis factor receptor superfamily, member 9
**G04**	Hs.478275	NM_003810	TNFSF10	Tumor necrosis factor (ligand) superfamily, member 10
**G05**	Hs.654445	NM_001244	TNFSF8	Tumor necrosis factor (ligand) superfamily, member 8
**G06**	Hs.437460	NM_000546	TP53	Tumor protein p53
**G07**	Hs.523968	NM_005426	TP53BP2	Tumor protein p53 binding protein, 2
**G08**	Hs.192132	NM_005427	TP73	Tumor protein p73
**G09**	Hs.460996	NM_003789	TRADD	TNFRSF1A-associated via death domain
**G10**	Hs.522506	NM_021138	TRAF2	TNF receptor-associated factor 2
**G11**	Hs.510528	NM_003300	TRAF3	TNF receptor-associated factor 3
**G12**	Hs.356076	NM_001167	XIAP	X-linked inhibitor of apoptosis

Table S2. Summarizes the effects of CK on apoptosis-related gene expression in K562 cells

		5 µM CK/Vehicle (comparing to vehicle)				5 µM CK/Vehicle (comparing to vehicle)	
Position	Symbol	p-value	Fold Change	Position	Symbol	p-value	Fold Change
A01	ABL1	0.078242	0.61	D07	CFLAR	0.750410	1.38
A02	AIFM1	0.382903	0.81	D08	CIDEA	0.178854	3.34
A03	AKT1	0.143119	1.72	D09	CIDEB	0.157238	2.98
A04	APAF1	0.139310	1.63	D10	CRADD	0.094013	1.68
A05	BAD	0.349755	1.48	D11	CYCS	0.254533	0.46
A06	BAG1	0.443388	1.27	D12	DAPK1	0.258410	1.38
A07	BAG3	0.023577	2.24	E01	DFFA	0.125697	1.74
A08	BAK1	0.141730	1.65	E02	DIABLO	0.789933	0.45
A09	BAX	0.222205	1.39	E03	FADD	0.009598	1.94
A10	BCL10	0.353384	1.26	E04	FAS	0.253858	1.58
A11	BCL2	0.013471	3.82	E05	FASLG	0.408652	1.63
A12	BCL2A1	0.181237	2.26	E06	GADD45A	0.112430	0.14
B01	BCL2L1	0.723142	1.28	E07	HRK	0.213097	1.69
B02	BCL2L10	0.028483	1.98	E08	IGF1R	0.002951	2.26
B03	BCL2L11	0.331363	1.35	E09	IL10	0.094532	2.76
B04	BCL2L2	0.620635	1.04	E10	LTA	0.486769	1.27
B05	BFAR	0.453454	1.73	E11	LTBR	0.666177	1.14
B06	BID	0.001721	2.00	E12	MCL1	0.042353	2.16
B07	BIK	0.854042	1.09	F01	NAIP	0.330213	1.18
B08	BIRC2	0.228796	1.78	F02	NFKB1	0.050347	2.25
B09	BIRC3	0.005223	5.18	F03	NOD1	0.288428	1.10
B10	BIRC5	0.069186	1.95	F04	NOL3	0.470623	1.23
B11	BIRC6	0.414949	1.15	F05	PYCARD	0.017232	3.82
B12	BNIP2	0.099144	1.59	F06	RIPK2	0.078172	1.86
C01	BNIP3	0.099902	1.67	F07	TNF	0.303590	1.66
C02	BNIP3L	0.689074	0.94	F08	TNFRSF10A	0.000736	2.06
C03	BRAF	0.106521	1.68	F09	TNFRSF10B	0.031042	1.77
C04	CASP1	0.852432	1.19	F10	TNFRSF11B	0.029121	2.89
C05	CASP10	0.029024	2.14	F11	TNFRSF1A	0.171394	1.82
C06	CASP14	0.098669	2.96	F12	TNFRSF1B	0.017493	2.61
C07	CASP2	0.024532	1.93	G01	TNFRSF21	0.260282	1.38
C08	CASP3	0.076737	1.94	G02	TNFRSF25	0.024494	2.36
C09	CASP4	0.968248	0.82	G03	TNFRSF9	0.006308	10.77
C10	CASP5	0.392628	1.44	G04	TNFSF10	0.869217	1.13
C11	CASP6	0.208489	1.64	G05	TNFSF8	0.172896	2.29
C12	CASP7	0.194797	1.41	G06	TP53	0.039596	1.66
D01	CASP8	0.082055	1.62	G07	TP53BP2	0.348832	0.70
D02	CASP9	0.058559	1.71	G08	TP73	0.003894	2.47
D03	CD27	0.020520	2.38	G09	TRADD	0.365561	1.69
D04	CD40	0.074750	2.07	G10	TRAF2	0.123259	1.56
D05	CD40LG	0.177088	2.87	G11	TRAF3	0.002306	2.84
D06	CD70	0.434469	0.99	G12	XIAP	0.374691	1.32

Notes: Listed are official gene symbols representing the mean fold-change in gene expression induced by a CK relative to the vehicle group. Red is up-regulated genes (at least 2.5 fold)

Table S3. Summarizes the effects of CK on apoptosis-related gene expression in Meg-01 cells

		5 µM CK/Vehicle (comparing to vehicle)				5 µM CK/Vehicle (comparing to vehicle)	
Position	Symbol	p-value	Fold Change	Position	Symbol	p-value	Fold Change
A01	ABL1	0.987296	0.30	D07	CFLAR	0.453888	1.33
A02	AIFM1	0.252329	1.27	D08	CIDEA	0.36335	2.24
A03	AKT1	0.42639	3.51	D09	CIDEB	0.425517	1.53
A04	APAF1	0.287746	1.29	D10	CRADD	0.249983	2.99
A05	BAD	0.159705	1.28	D11	CYCS	0.594734	0.84
A06	BAG1	0.192988	1.24	D12	DAPK1	0.14568	1.62
A07	BAG3	0.817328	2.75	E01	DFFA	0.766128	0.98
A08	BAK1	0.964686	0.97	E02	DIABLO	0.946968	0.97
A09	BAX	0.353756	0.41	E03	FADD	0.233841	0.23
A10	BCL10	0.517713	0.46	E04	FAS	0.766639	0.63
A11	BCL2	0.470415	2.44	E05	FASLG	0.238575	1.43
A12	BCL2A1	0.3165	1.44	E06	GADD45A	0.008744	5.76
B01	BCL2L1	0.547207	0.34	E07	HRK	0.878739	2.38
B02	BCL2L10	0.510969	1.29	E08	IGF1R	0.477029	2.95
B03	BCL2L11	0.118561	1.30	E09	IL10	0.793512	0.63
B04	BCL2L2	0.063053	2.97	E10	LTA	0.08103	2.72
B05	BFAR	0.031103	3.70	E11	LTBR	0.847754	0.28
B06	BID	0.003165	0.23	E12	MCL1	0.935635	0.98
B07	BIK	0.361254	1.52	F01	NAIP	0.195774	1.34
B08	BIRC2	0.044983	3.71	F02	NFKB1	0.244557	3.00
B09	BIRC3	0.928447	1.08	F03	NOD1	0.543145	1.15
B10	BIRC5	0.27492	3.96	F04	NOL3	0.37791	1.99
B11	BIRC6	0.140901	1.42	F05	PYCARD	0.458198	1.64
B12	BNIP2	0.50823	1.24	F06	RIPK2	0.837534	0.91
C01	BNIP3	0.092282	0.28	F07	TNF	0.466736	0.43
C02	BNIP3L	0.056271	1.87	F08	TNFRSF10A	0.914914	0.90
C03	BRAF	0.67985	0.86	F09	TNFRSF10B	0.496213	1.12
C04	CASP1	0.074841	1.72	F10	TNFRSF11B	0.057733	2.06
C05	CASP10	0.935901	0.83	F11	TNFRSF1A	0.41792	1.15
C06	CASP14	0.571604	0.94	F12	TNFRSF1B	0.200407	0.75
C07	CASP2	0.772751	0.93	G01	TNFRSF21	0.174034	3.65
C08	CASP3	0.54747	1.07	G02	TNFRSF25	0.620967	1.01
C09	CASP4	0.506404	2.51	G03	TNFRSF9	0.71134	0.53
C10	CASP5	0.280794	0.37	G04	TNFSF10	0.920045	1.41
C11	CASP6	0.023471	4.26	G05	TNFSF8	0.576348	1.21
C12	CASP7	0.585525	1.17	G06	TP53	0.580887	1.33
D01	CASP8	0.374794	1.20	G07	TP53BP2	0.96011	1.11
D02	CASP9	0.627295	1.05	G08	TP73	0.730711	1.91
D03	CD27	0.817434	0.98	G09	TRADD	0.624757	0.57
D04	CD40	0.556961	0.73	G10	TRAF2	0.046017	3.94
D05	CD40LG	0.941153	0.79	G11	TRAF3	0.453782	2.40
D06	CD70	0.613071	0.67	G12	XIAP	0.156215	3.90

Notes: Listed are official gene symbols representing the mean fold-change in gene expression induced by a CK relative to the vehicle group. Red is up-regulated genes (at least 2.5 fold)
