# Supplementary figures and images for: Compound K Promotes Megakaryocytic Differentiation by NLRP3 Inflammasome Activation
Source: Biomolecules. 2024 Oct 4;14(10):1257. doi: 10.3390/biom14101257 (PMC11506438; doi:10.3390/biom14101257)

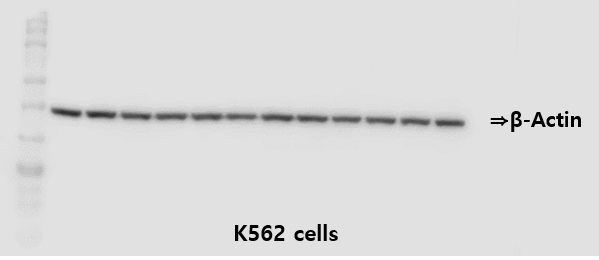

Supplement: Supplementary file 1 [file biomolecules-14-01257-s001.zip › Original Images/K562 cells a--Actin(1).png]

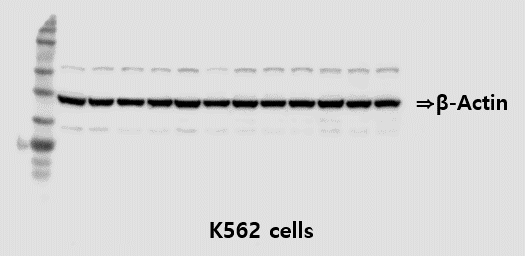

Supplement: Supplementary file 1 [file biomolecules-14-01257-s001.zip › Original Images/K562 cells a--Actin(2).png]

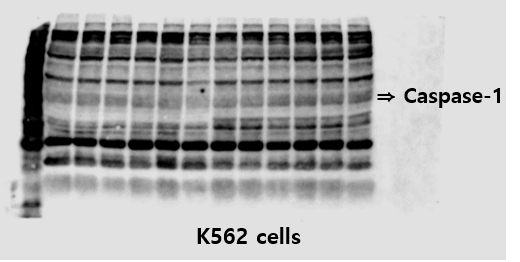

Supplement: Supplementary file 1 [file biomolecules-14-01257-s001.zip › Original Images/K562 cells Caspase-1.png]

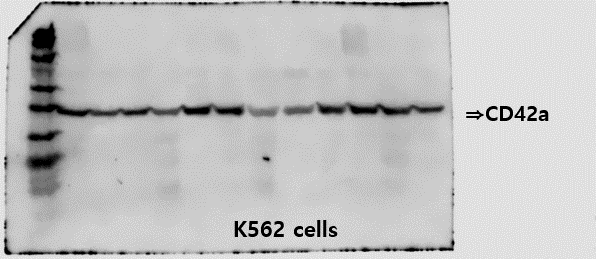

Supplement: Supplementary file 1 [file biomolecules-14-01257-s001.zip › Original Images/K562 cells CD42a.png]

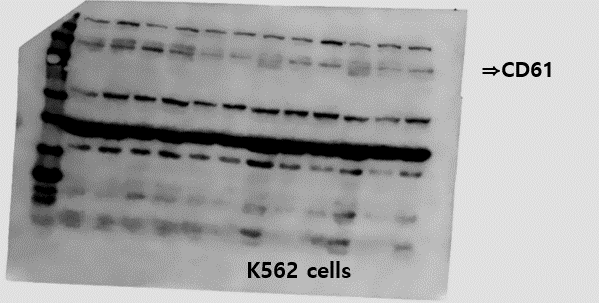

Supplement: Supplementary file 1 [file biomolecules-14-01257-s001.zip › Original Images/K562 cells CD61.png]

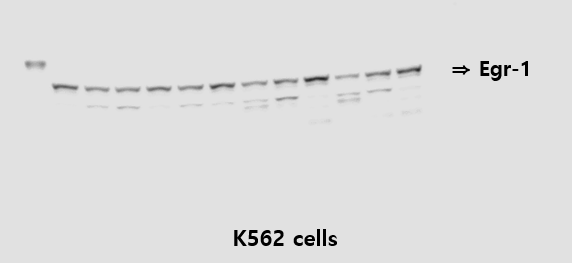

Supplement: Supplementary file 1 [file biomolecules-14-01257-s001.zip › Original Images/K562 cells Egr-1.png]

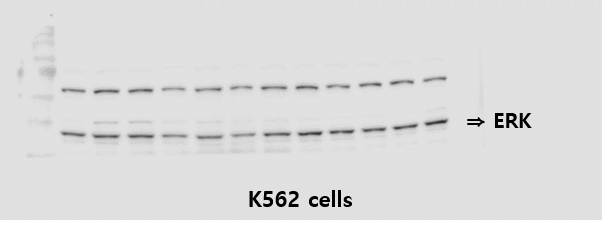

Supplement: Supplementary file 1 [file biomolecules-14-01257-s001.zip › Original Images/K562 cells ERK.png]

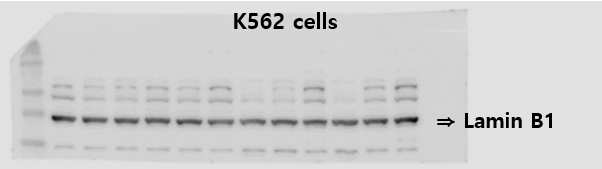

Supplement: Supplementary file 1 [file biomolecules-14-01257-s001.zip › Original Images/K562 cells Lamin B1.png]

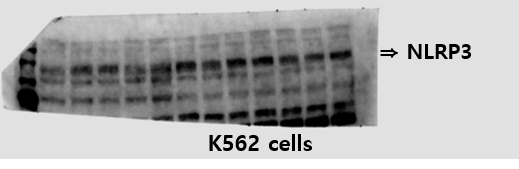

Supplement: Supplementary file 1 [file biomolecules-14-01257-s001.zip › Original Images/K562 cells NLRP3.png]

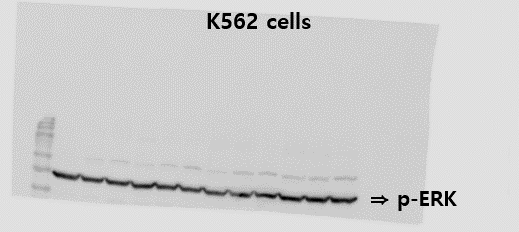

Supplement: Supplementary file 1 [file biomolecules-14-01257-s001.zip › Original Images/K562 cells p-ERK.png]

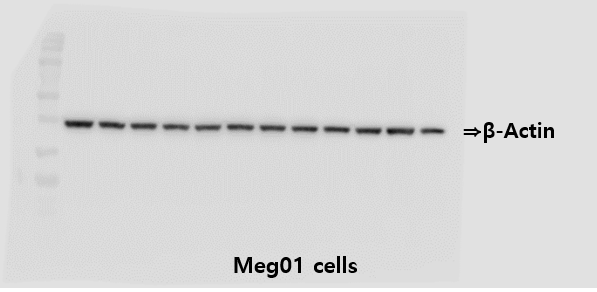

Supplement: Supplementary file 1 [file biomolecules-14-01257-s001.zip › Original Images/Meg01 cells a--Actin(1).png]

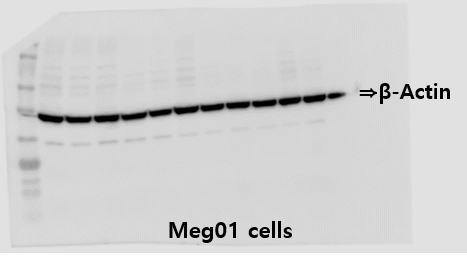

Supplement: Supplementary file 1 [file biomolecules-14-01257-s001.zip › Original Images/Meg01 cells a--Actin(2).png]

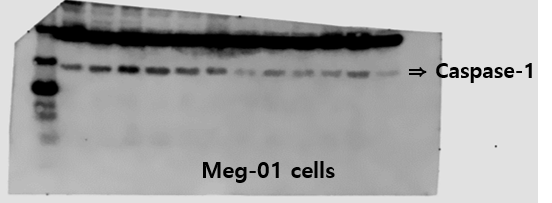

Supplement: Supplementary file 1 [file biomolecules-14-01257-s001.zip › Original Images/Meg01 cells Caspase-1.png]

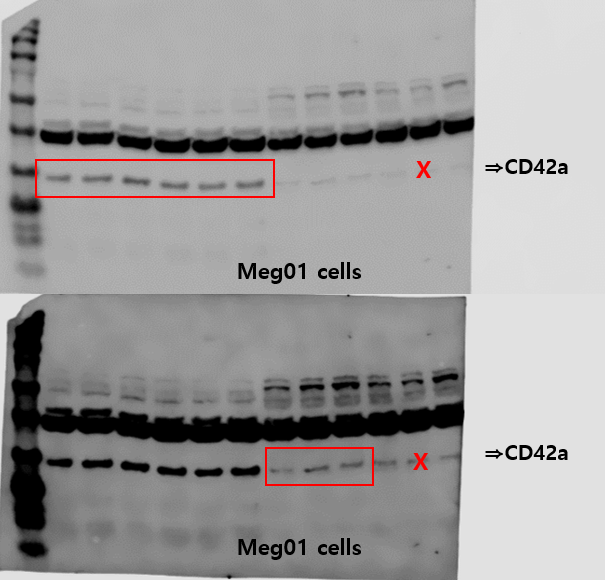

Supplement: Supplementary file 1 [file biomolecules-14-01257-s001.zip › Original Images/Meg01 cells CD42a.png]

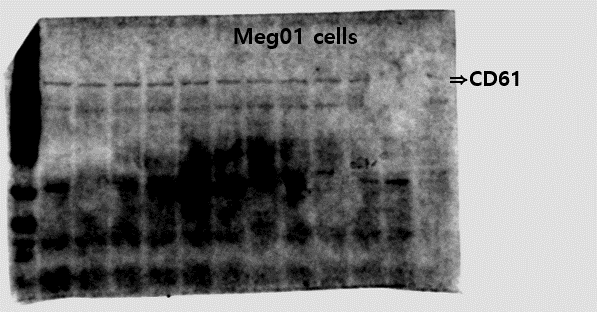

Supplement: Supplementary file 1 [file biomolecules-14-01257-s001.zip › Original Images/Meg01 cells CD61.png]

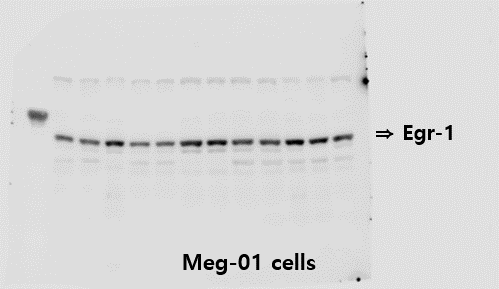

Supplement: Supplementary file 1 [file biomolecules-14-01257-s001.zip › Original Images/Meg01 cells Egr-1.png]

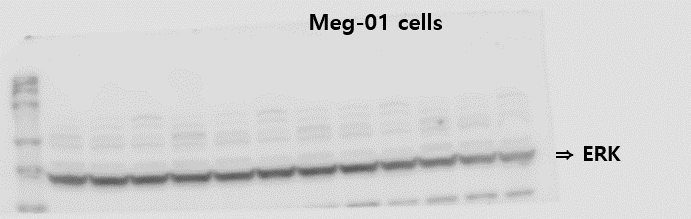

Supplement: Supplementary file 1 [file biomolecules-14-01257-s001.zip › Original Images/Meg01 cells ERK.png]

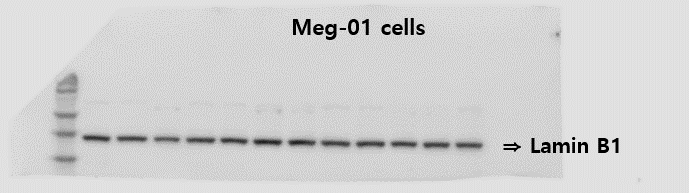

Supplement: Supplementary file 1 [file biomolecules-14-01257-s001.zip › Original Images/Meg01 cells Lamin B1.png]

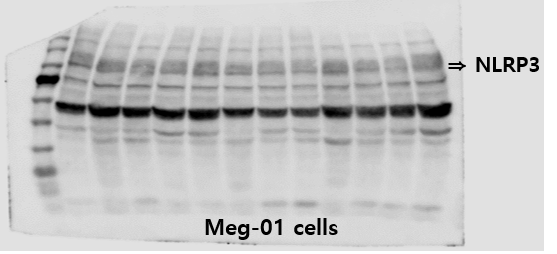

Supplement: Supplementary file 1 [file biomolecules-14-01257-s001.zip › Original Images/Meg01 cells NLRP3.png]

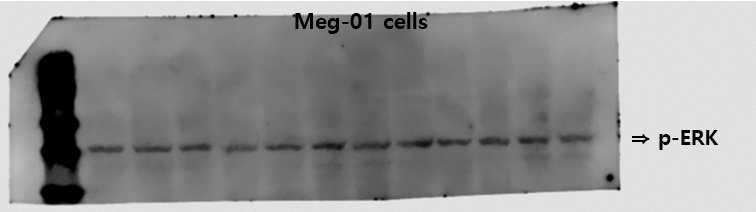

Supplement: Supplementary file 1 [file biomolecules-14-01257-s001.zip › Original Images/Meg01 cells p-ERK.png]
